# Pet Ownership and Evacuation Prior to Hurricane Irene

**DOI:** 10.3390/ani2040529

**Published:** 2012-09-28

**Authors:** Melissa G. Hunt, Kelsey Bogue, Nick Rohrbaugh

**Affiliations:** Department of Psychology, University of Pennsylvania, 3720 Walnut St., Philadelphia, PA 19104, USA; E-Mails: kbogue@sas.upenn.edu (K.B.); nrohr@sas.upenn.edu (N.R.)

**Keywords:** pet ownership, evacuation failure, emergency, shelter, psychopathology

## Abstract

**Simple Summary:**

Ninety pet owners and 27 non-pet owners who lived in mandatory evacuation zones during the 2011 Hurricane Irene were surveyed about whether or not they evacuated and about their experiences during the hurricane. Although pet-ownership was not statistically associated with evacuation failure, many pet owners who chose not to evacuate still claimed that they did not evacuate because of difficulties with evacuating their pet. These findings suggest that more work needs to be done in order to make evacuating with a pet easier.

**Abstract:**

Pet ownership has historically been one of the biggest risk factors for evacuation failure prior to natural disasters. The forced abandonment of pets during Hurricane Katrina in 2005 made national headlines and led to the passage of the Pet Evacuation and Transportation Standards Act (PETS, 2006) which mandated local authorities to plan for companion animal evacuation. Hurricane Irene hit the East Coast of the United States in 2011, providing an excellent opportunity to examine the impact of the PETS legislation on frequency and ease of evacuation among pet owners and non-pet owners. Ninety pet owners and 27 non-pet owners who lived in mandatory evacuation zones completed questionnaires assessing their experiences during the hurricane and symptoms of depression, PTSD, dissociative experiences, and acute stress. Pet ownership was not found to be a statistical risk factor for evacuation failure. However, many pet owners who failed to evacuate continue to cite pet related reasons.

## 1. Introduction

Evacuation failure during an emergency is a common problem that can have disastrous consequences for individuals and public safety personnel. By choosing not to evacuate one’s home even when ordered to do so by local government, many people put themselves at risk of injury or death during an emergency, as well as psychological trauma that may result from exposure to the emergency in an unsafe environment. Evacuation failure also puts other people at risk, since first responders to the emergency are also put in danger as they go to rescue those who failed to evacuate. Heath and his colleagues [1,2] have identified several risk factors for household evacuation failure. One study conducted after mandatory evacuation orders were issued due to flooding in Yuba County, California found that the largest contributing factor to compliance with evacuation orders was having children in the house—households with children were twice as likely to evacuate as households without children. Factors that were found to be associated with increased evacuation failure included higher level of education of the head of household (OR = 1.8), higher age of the head of household (OR = 1.02), and owning more dogs (OR = 1.5) or cats (OR = 1.2) [2].

Another study following the same natural disaster examined why some families evacuated themselves but chose to leave their pets behind. Low pet attachment was associated with an increased risk of evacuation failure. The two most common reasons given for not evacuating pets were that the owners did not believe they would be gone for long, and that the greatest concern was for the safety of family members. However, several other common responses showed that many pets were left behind during evacuation due to poor emergency planning. Inability to transport a pet during an emergency and lack of knowledge of pet-friendly emergency shelters were popular explanations for pet evacuation failure. Many pet owners in the study made arrangements to have their pets taken care of by others who had chosen not to evacuate, quite possibly solidifying their dangerous choice to remain in the area [1]. In addition, people returning prematurely on-site to rescue animals that were initially left behind places both the individuals and first responders at risk. 

After Hurricane Katrina affected much of the southeastern United States in August 2005, more research was published that focused on the effects of natural disasters on companion animals and their owners. Hurricane Katrina was particularly devastating for cats and dogs in the region. Estimating the number of companion animals lost to death or displacement during the hurricane is a difficult task, as some states in the area do not require veterinary licensing and therefore it is impossible to know how many animals were treated at these unlicensed clinics. However, online resources like Petfinder.com listed more than 17,000 found animals and handled 22,000 rescue requests in the aftermath of the storm, suggesting at least 40,000 to 50,000 animals lost [3]. The poor conditions following the storm coupled with an abundance of lost or stray animals led to the spread of multiple infectious diseases in the area. Many lost animals treated by veterinary clinics in the region were never matched with their previous owners and were thus sent across the country to various rescue and adoption agencies. Even more animals were moved across the United States due to the displacement of their owners after the storm. This translocation of many possibly infected companion animals may have contributed to increased rates of infection in geographic areas where these infections were not previously prevalent [4].

A Gallup survey of Hurricane Katrina survivors identified several factors that were associated with increased risk of experiencing pet loss during the storm. African-Americans were more likely to experience pet loss than whites, and younger people were more likely to experience pet loss than their older counterparts. Even more surprising is the finding that those who evacuated were 1.65 times more likely to lose a pet during the storm than those who did not evacuate, showing clear evidence of why people may fail to evacuate in order to protect their pets in the face of poor evacuation planning [5].

Further research has shown that pet loss during natural disasters can be psychologically traumatic. Hunt, Al-Awadi, and Johnson [6] investigated signs of depression, PTSD, acute stress, pet attachment and bereavement, and dissociative experiences in pet owners who had been affected by Hurricane Katrina. Results showed that while some demographic differences such as marital status and level of education did have effects on psychological outcomes, losing one’s pet had the greatest effect. People who had lost their animal companion due to the hurricane had similar levels of psychopathology as people who had lost their homes. In some cases, pet loss was a better predictor of psychopathology than home loss, as pet loss resulted in higher levels of PTSD and depression than did home displacement, although both caused acute stress. Surprisingly, people who lost both their home and their pet had similar psychopathological outcomes as those who lost only their pets [6].

Following widespread negative media coverage of the inability of residents of New Orleans to evacuate their pets during Hurricane Katrina, the U.S. Congress passed the Pet Evacuation and Transportation Standards (PETS) Act, signed into law by President Bush on 6 October 2006 [7]. The law allowed the Federal Emergency Management Agency (FEMA) to aid state and local governments in creating evacuation plans for companion and service animals. The PETS Act also allowed federal funds to be administered in order to build emergency shelters for pets [8]. However, the impact of the legislation has since been questioned. An informal survey by the American Kennel Club in May 2006 [9], only a few months after Hurricane Katrina, found that 62% of respondents said they would defy mandatory evacuation orders in order to remain with their pets during a hypothetical emergency. Over half of the respondents in the survey who did not have an evacuation plan for their pet said that they would like to construct such a plan but did not know how [9]. A recent survey of animal care and control agencies in Ohio also found that many of these agencies are not properly prepared to respond to an emergency. Only 49% of agencies that responded were in contact with other agencies regarding emergency and disaster response planning. Only 12% of these agencies had developed a written plan to respond to emergencies, and another 19% claimed to be in the process of developing such a plan. Particularly troubling is the finding that only one third of the agencies that responded to the survey were even aware of the PETS Act of 2006 [10].

In light of the passage of the PETS Act and allegations of its impact as questionable, the aims of our current study were threefold: First, we attempted to identify any demographic risk factors for increased evacuation failure in a similar manner as Heath *et al.* [1,2]. Second, we tried to gain a clear picture of the experience of evacuating one’s pet following the passage and implementation of the PETS Act of 2006. Finally, we seek to re-examine the psychological impact of natural disasters and discover any significant differences between pet owners and non-pet-owners, evacuees and those who failed to evacuate, those who lost their pets and/or experienced damage to their homes, and across any of the other variables for which we collected data.

## 2. Experimental Section

### 2.1. Procedure

Participants were 90 pet owners and 27 non-pet owners who lived in mandatory evacuation zones during Hurricane Irene, including North Carolina (n = 43), New Jersey (n = 37), Maryland (n = 12), Pennsylvania (n = 11), New York (n = 8), Delaware (n = 3), Connecticut (n = 1), Virginia (n = 1), and Vermont (n = 1). The sample was well balanced with respect to gender (53% female, 47% male). Participants covered a wide range of ages (M = 43, SD = 15, range 18–87). They were mostly married (53%) or single (35%) with the remainder of the sample being divorced (8%), separated (2%) or widowed (2%). Less than half of the households represented reported having one child or more (36%). The vast majority of the sample was white (96%) with only single participants reporting that they were Asian, black, mixed race or not reporting. The sample was relatively well-off economically, with 5% of the sample each reporting household incomes of less then $20,000, less than $30,000 and less than $40,000. Approximately 35% of the sample reported household incomes between $40,000 and $75,000 per year. Finally, almost exactly half of the sample (50%) reported annual household incomes greater than $75,000.

Of the 90 pet-owners in our study, 69 participants (77%) reported owning at least one dog and 51 participants (57%) reported owning at least one cat. Nine participants (10%) reported owning fish, 2 participants (2%) reported owning parakeets, and only one participant (1%) reported owning each of the following: frogs, chickens, snakes, hamsters, and ferrets. No participants reported owning a pet other than a dog or a cat who did not also own a dog or a cat. Participants were given surveys where the specific questions varied based on two dimensions: those who were pet-owners *versus* those who were non pet-owners and those who evacuated *versus* those who did not evacuate. 

To attract the most representative sample possible, many different avenues were utilized to invite potential participants to the study. An invitational message was posted on a number of internet message boards, including www.craigslist.com, www.obxconnection.com, and www.tripadvisor.com. A Facebook profile was also created to post on various Facebook pages, such as those for PetSmart, Hurricane Irene, and the New Jersey Shore. Local classified ads were printed in papers in affected coastal communities in New Jersey and North Carolina for one to two weeks and residents of these areas were also contacted via randomized telephone calls and asked to participate in the study. Paper flyers were mailed to agreeable veterinary offices in affected areas and were posted by the offices in visible locations. Participants were also approached in person in the Atlantic City Train Station to fill out a paper survey.

The invitation to the study informed the viewer that the study was about people’s experiences during Hurricane Irene as well as the people’s emotional aftereffects following the hurricane. The link within the message led them to the study website, where they were consented and invited to complete a battery of questionnaires. Those participating over the telephone verbally consented to the study and those approached in person consented with their signature.

### 2.2. Materials

#### 2.2.1. Demographic Descriptors and Hurricane Experiences Questionnaire

Questions designed to ascertain demographic characteristics of the respondents included age, city and state of residence, gender, race, marital status, number of children, education level, and income level. Additionally, questions concerning human and material loss as a result of Hurricane Irene, as well as questions about pet ownership were posed. All participants were asked about the extent to which certain factors influenced their decision to evacuate or fail to evacuate. Questions about the evacuation site and overall evacuation experience were also posed to those who did evacuate. Pet owners were asked specific questions about the experience of evacuating with a pet, if applicable. 

#### 2.2.2. Beck Depression Inventory (BDI-II) [11,12]

This 21-item, forced-choice questionnaire measures depressive symptoms. Items are scored on a 0–3 scale and summed to produce a total score, with higher scores indicating more depressive symptoms. This measure has moderate to good internal consistency and good test-retest reliability [12]. For the BDI-II, Beck *et al.* [12] suggest that symptom scores of 0–13 be interpreted as minimal, 14–19 as mild, 20–28 as moderate, and 29–63 as severe. In this study, question 9, concerning suicidal ideation, was removed.

#### 2.2.3. PTSD Symptom Scale Self-Report (PSS-SR) [13]

This questionnaire consists of the 17 symptoms from the DSM-IV [14] description of PTSD. Respondents are asked to complete PSSS-R items by rating the frequency of each symptom over the last week on a scale from 0 (not at all) to 4 (almost always). The highest possible score is 68. 

#### 2.2.4. Peri-Traumatic Dissociative Experiences Questionnaire–Self-Report (PDEQ-SR) [15]

This self-report, 10-item questionnaire asks respondents to what extent they experienced depersonalization, derealization, confusion, concentration and memory deficits during and immediately following the time of the traumatic event. Dissociative symptoms include feeling as if events are not real, but more like a movie or dream; feeling as if one were a spectator observing oneself; experiencing changes in time sense, especially as if things were happening in slow motion; and experiencing confusion, periods of “blankness” or lack of awareness, and a feeling of disconnection from one’s body, surroundings, or emotional experience. Peri-traumatic symptoms are measured on a 5-point scale from 1 (not true at all) to 5 (extremely true). The highest possible score is 50.

#### 2.2.5. Stanford Acute Stress Reaction Questionnaire (SARSQ) [16]

This questionnaire consists of 10 items from the DSM-IV [14] description of Acute Stress Disorder, asking respondents if they have experienced numbing, detachment, de-realization, depersonalization, or dissociative amnesia. Respondents are asked to what extent they experienced these symptoms during and immediately after a disaster. They rate their reaction on a 7-point scale from 0 (not experienced) to 6 (very often experienced). The highest possible score is 60. 

#### 2.2.6. Pet Attachment Questionnaire (PAQ) [17]

This questionnaire is an 8-item questionnaire devised to assess pet attachment. It has good internal consistency (α = 0.75 in [6]) and good factor structure. Stallones *et al.* [17] found the questionnaire to be appropriate for all companion animals, not only cats and dogs. Pet attachment has been found to be correlated with degree of grief after pet loss [18]. 

## 3. Results

### 3.1. Pet Ownership as a Risk Factor for Evacuation Failure

**Figure 1:**
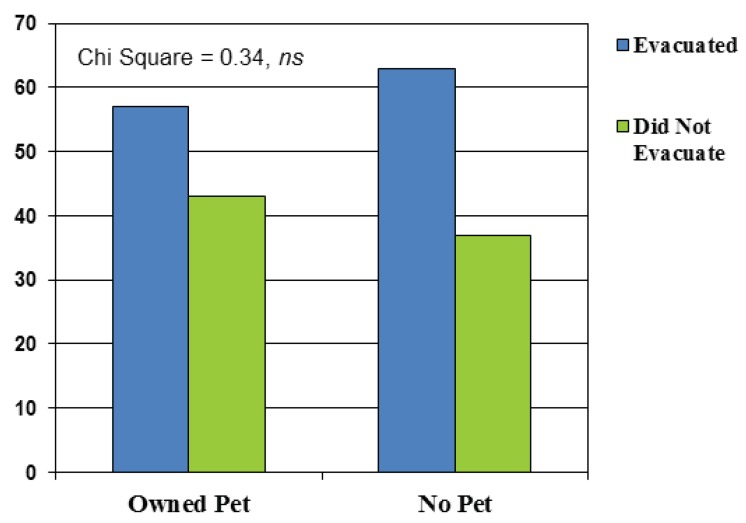
Percent of households evacuating among pet owners and non-pet owners.

Owning a pet did not emerge as a statistically significant risk factor for failing to evacuate prior to Hurricane Irene in this sample. Of the pet owners in our sample, 51 (57%) evacuated and 39 (43%) did not evacuate. Of the non-pet-owners in our sample, 17 (63%) evacuated and 10 (37%) did not evacuate (χ² = 0.34, ns). See Figure 1. Owning a pet other than a dog or cat was also unrelated to evacuation choices (χ² = 1.2, ns). 

### 3.2. Reasons for Failing to Evacuate Among Pet Owners

**Figure 2:**
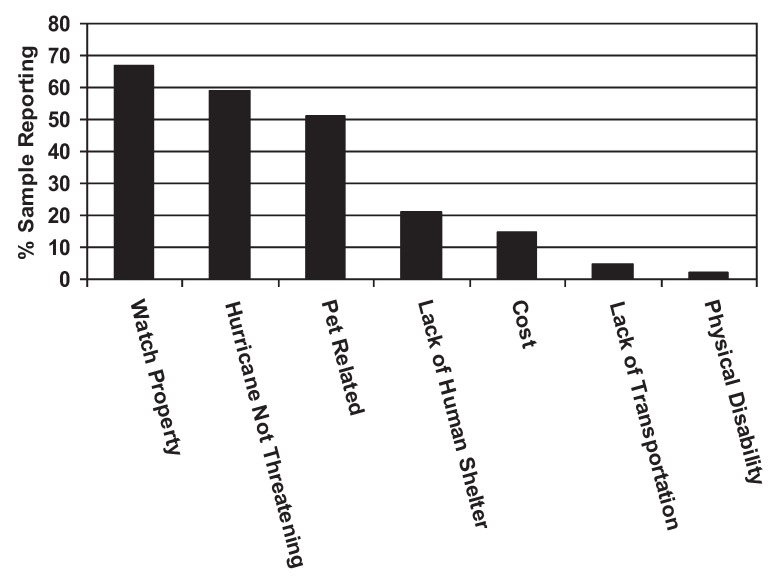
Reasons for Evacuation Failure by Pet Owners.

While owning a pet was not a statistically significant risk factor for evacuation failure in our sample, twenty pet owners (51%) who failed to evacuate endorsed pet-related factors as influencing their evacuation decisions to a great degree. These factors included things like inability to transport pet, cost of transporting or sheltering pet, and lack of a place for the pet to stay. Twenty-six pet owners who did not evacuate said that wanting to watch their property was the single most important factor in their decision to not evacuate. Twenty-three pet owners said they felt the hurricane was nonthreatening. Other primary reasons for not evacuating among pet owners included lack of shelter for people (8 people), cost of evacuating (6 people), physical disability (2 people), and lack of transportation (1 person). See Figure 2. 

### 3.3. Locations Where People Sheltered

The majority of our evacuating sample (71%) stayed with a friend or family member. Only 6% of the sample reported staying at an emergency evacuation shelter during the storm. Fourteen percent of the sample evacuated to a hotel, and 8% of the sample responded “Other” to the question about where they evacuated. 

### 3.4. Difficulty Evacuating

**Figure 3:**
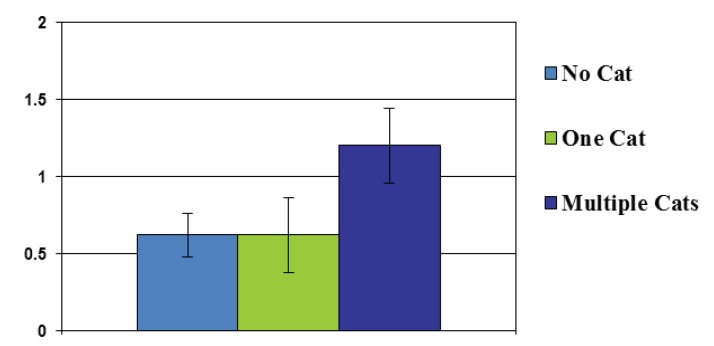
Difficulty evacuating among non-cat owners, owners of one cat, and owners of multiple cats.

Owning a dog, the number and size of dogs in a household, and owning a cat were not associated with failing to evacuate or difficulty evacuating. The number of cats owned, however, was associated with increased difficulty evacuating. Actual number of cats owned was highly correlated with evacuation difficulty (*r *= 0.61, *p *< 0.001). Moreover, individuals who owned multiple (2 or more) cats rated evacuating as significantly more difficult than individuals with no cats, or only 1 cat (F(2,41) = 16.01, *p *< 0.001). See Figure 3.

### 3.5. Failing to Evacuate Pets

Among pet owners who themselves evacuated, only 2 individuals reported failing to evacuate their pets. While this sample size is too small to conduct meaningful inferential statistics, the mean level of pet attachment reported by these individuals (M = 8, SD = 2.8) was substantially lower than that reported by individuals who did evacuate their pets (M = 15, SD = 4.3).

### 3.6. Psychopathology

**Figure 4:**
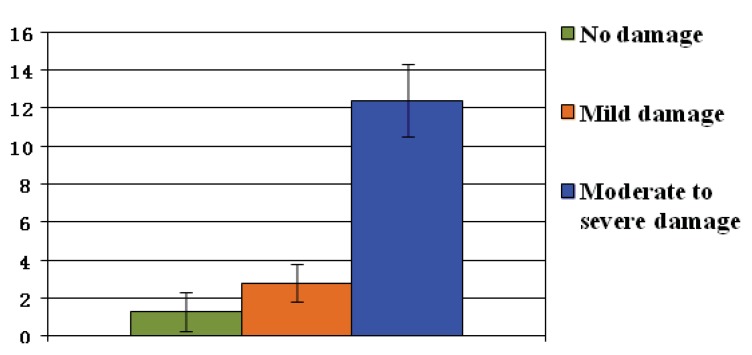
Symptoms of acute stress by level of property damage.

No participants in our study lost a pet during Hurricane Irene. In addition, none of our participants reported loss of human life among their family or friends. Overall rates of symptoms of psychopathology were fairly low (BDI: M = 6, SD = 7.6; PSSSR: M = 2.8, SD = 5; PDEQ: M = 12, SD = 6.7; SASRQ: M = 3.4, SD = 7). Two conservative omnibus MANOVAs were carried out predicting psychopathology symptoms by pet ownership and by evacuation status. Neither was significant. All psychopathology scales were significantly correlated with each other (all *r *> 0.23, all *p *< 0.05). Only two significant associations between hurricane specific factors and psychopathology emerged. First, acute stress, peri-traumatic dissociation and PTSD symptoms were all positively correlated with level of property damage (all *r *> 0.30, all *p *< 0.01). Second, the individuals who reported experiencing injury during the hurricane reported significantly higher levels of clinical distress across all measures (all t(94) > 2.3, all *p *< 0.05). See Figure 4.

## 4. Discussion

Overall, we found that owning a pet was not a statistically significant risk factor for evacuation failure prior to Hurricane Irene. This suggests that the media coverage around Hurricane Katrina and the subsequent PETS legislation have had positive effects on the evacuation of animals and that general awareness about the importance of evacuating pets has increased significantly as a result of the devastation of Hurricane Katrina. Because of the mechanisms put into place to aid with evacuations, this trend of improved evacuation compliance among pet owners will hopefully continue in future mandatory evacuation notices. 

However, pet owners who chose not to evacuate despite mandatory evacuation notices still cited pet related reasons for their evacuation failure. Indeed, 23% of the pet owners who did not evacuate cited pet related reasons as one of the primary causes of their evacuation failure. While many pet owners did evacuate their pets, it is unclear how many of these evacuators would have defied the evacuation laws if they could not have brought their pets. Although pet ownership did not statistically predict evacuation failure, it is clear that for many, the sheltering of a pet, the transportation of a pet, and/or the cost of evacuating the pet are still significant barriers to the evacuation of both pets and their owners.

Even though pet owners did evacuate at the same rate as non-pet-owners, pet-owners with two or more cats cited significant difficulty in evacuation over and above the difficulties reported by dog owners, single cat owners, and non-pet owners. This suggest a unique challenge presented to individuals trying to evacuate with multiple cats, as they must plan for extra crates and litter boxes in addition to the usual food and water supplies. One participant explicitly told us that after 10 h in the car with three howling cats stuck in traffic and waiting for ferries, they would not choose to evacuate again. Thus, because evacuation was difficult for individuals with multiple cats, they are also at risk for evacuation failure in the future, and municipalities may want to pay special attention to these particular pet owners in the future. 

Although all local municipalities now must have pet-friendly shelters in place to help evacuate animals as a result of the PETS Act, most individuals in our sample who evacuated, both with and without pets, did not take advantage of these shelters. Approximately 70% of all evacuators chose to shelter with a friend or family, as opposed to only 6% who chose to shelter at an emergency evacuation shelter. This may have to do with the relative affluence of our sample. They had multiple social resources and access to cars to transport themselves (and potentially their pets) and did not require the use of local shelters. However, a number of individuals did not evacuate because they stated that they did not know where to go with their pets. Informational interviews with many local authorities and municipalities indicated that responsible individuals in those municipalities had often taken great pains to stock crates, water bowls and emergency pet food supplies. While pets might not have been happy spending hours in stacked crates in elementary school hallways, they would certainly have been safe. In the future, local governments should advertise the locations and benefits of an evacuation shelter more effectively to teach citizens about their evacuation options and hopefully increase attendance at these shelters. 

The data also suggest that as a whole, Hurricane Irene was not perceived to be nearly as threatening or as dangerous as Hurricane Katrina. As the primary reason for evacuation failure, many participants cited that they felt the hurricane was simply non-threatening. Despite this, some individuals did experience mild to moderate levels of acute stress and peri-traumatic dissociation and were reporting mild to moderate levels of PTSD symptoms associated with property damage and personal injury. Thus, even natural disasters that are perceived in advance to be non-threatening can indeed be quite dangerous and can result in negative psychological sequelae. 

There were a number of important limitations to this study that impact the generalizability of the findings. First and foremost, our sample size was quite small and not particularly representative. Despite our efforts to use multi-modality recruitment strategies, we had a total sample of only 117 participants, and of those only 27 were non-pet owners. We were more successful with our recruitment efforts of pet owners (through flyers in vet’s offices and through web postings) than with non-pet owners. Second, the sample was unusually affluent and predominantly white. Demographic findings in the aftermath of Hurricane Katrina suggested that less affluent and minority individuals bore the brunt of the negative sequelae, and had fewer resources available to them to aid in successful evacuation. 

## 5. Conclusions

Overall, the data suggest that the PETS legislation and general hurricane awareness has had some impact on reducing evacuation failure secondary to pet ownership. Still, many pet owners cited pet related reasons for failing to evacuate or experienced significant confusion about, or difficulty with, evacuating their pets. Improvements can still be made to create an easier, more efficient pet evacuation process so that pet owners will continue to evacuate in the future. 

## References

[1] Heath S.E., Beck A.M., Kass P.H., Glickman L.T. (2001). Risk factors for pet evacuation failure after a slow-onset disaster. J. Am. Vet. Med. Assoc..

[2] Heath S.E., Kass P.H., Beck A.M., Glickman L.T. (2001). Human and pet-related risk factors for household evacuation failure during a natural disaster. Am. J. Epidemiol..

[3] Burns K. (2006). Summarizing a disaster, by the numbers. J. Am. Vet. Med. Assoc..

[4] Levy J.K., Lapin M.R., Glaser A.L., Birkenheuer A.J., Anderson T.C., Edinboro C.H. (2011). Prevalence of infectious diseases in cats and dogs rescued following Hurricane Katrina. J. Am. Vet. Med. Assoc..

[5] Zottarelli L.K. (2010). Broken bond: An exploration of human factors associated with companion animal loss during Hurricane Katrina. Sociol. Forum.

[6] Hunt M., Al-Awadi M., Johnson M. (2008). Psychological sequelae of pet loss following Hurricane Katrina. Anthrozoos.

[7] Pets Evacuation and Transportation Standards Act of 2006. http://www.thomas.gov/cgi-bin/bdquery/z?d109:h.r.03858.

[8] Nolen R.S. (2006). Congress orders disaster planners to account for pets. J. Am. Vet. Med. Assoc..

[9] American Kennel Club. AKC survey finds majority of owners would defy emergency evacuation orders and stay with pets. http://www.akc.org/pdfs/press_center/press_releases/2006/Hurricane_Survey.pdf.

[10] Decker S.M., Lord L.K., Walker W.L., Wittum T.E. (2010). Emergency and disaster planning at Ohio animal shelters. J. Appl. Anim. Welf. Sci..

[11] Beck A.T., Ward C.H., Mendelson M., Mock J., Erbaugh J. (1961). An inventory for measuring depression. Arch. Gen. Psych..

[12] Beck A.T., Steer R.A., Brown G.K. (1996). Manuel for the Beck Depression Inventory.

[13] Foa E.B., Riggs D.S., Dancu C.V., Rothbaum B.O. (1993). Reliability and validity of a brief instrument for assessing post-traumatic stress disorder. J. Trauma. Stress.

[14] (2000). Diagnostic and Statistical Manual of Mental Disorders: DSM-IV-TR.

[15] Marmar C.R., Metzler T.J., Otte C., Wilson J.P., Keane T.M. (1997). The peritraumatic dissociative experiences questionnaire. Assessing Psychological Trajuma and PTSD.

[16] Cardena E., Classen C., Spiegel D. (1991). Standford Acute Stress Reaction Questionnaire.

[17] Stallones L., Johnson T.P., Garrity T.F., Marx M.B. (1990). Quality of attachment to companion animals among U.S. adults 21 to 64 years of age. Anthrozoos.

[18] Hunt M., Padilla Y. (2006). Development of the Pet Bereavement Questionnaire. Anthrozoos.

